# Supplementary material for: Function and firing of the Streptomyces coelicolor contractile injection system requires the membrane protein CisA
Source: eLife. 2025 Jul 8;14:RP104064. doi: 10.7554/eLife.104064 (PMC12237407; doi:10.7554/eLife.104064)
Supplement: Figure 3—source data 1. [file elife-104064-fig3-data1.docx]

**Figure 3-source data 1.** Proteins detected by mass spectrometry in samples of purified CIS^Sc^ after crude sheath preparation from a *S. coelicolor* non-contractile CIS^Sc^ mutant (*S. coelicolor* CIS-N5) without exogenous stress and following nisin stress.

| Protein ID | CIS ID | *S. coelicolor* CIS-N5 | |
| --- | --- | --- | --- |
|  |  | Without exogenous stress | Upon nisin stress |
| SCO4242 | CisA | - | 5% coverage / 2 total unique peptide |
| SCO4243 | Cis12 | 6% coverage / 1 total unique peptide | 5% coverage / 1 total unique peptide |
| SCO4244 | Cis11 | 13% coverage / 6 total unique peptide | 4% coverage / 2 total unique peptide |
| SCO4245 | Cis9 | 21% coverage / 2 total unique peptide | 20% coverage / 2 total unique peptide |
| SCO4246 | Cis8 | 18% coverage / 10 total unique peptide | 13% coverage / 7 total unique peptide |
| SCO4247 | Cis7 | 5% coverage / 1 total unique peptide | 4% coverage / 1 total unique peptide |
| SCO4248 | Cis1b | 22% coverage / 2 total unique peptide | 20% coverage / 2 total unique peptide |
| SCO4249 | - | 5% coverage / 1 total unique peptide | 4% coverage / 1 total unique peptide |
| SCO4252 | Cis1a | 26% coverage / 4 total unique peptide | 19% coverage / 3 total unique peptide |
| SCO4253 | Cis2 | 23% coverage / 9 total unique peptide | 30% coverage / 14 total unique peptide |
| SCO4254 | - | 10% coverage / 6 total unique peptide | 10% coverage / 6 total unique peptide |
| SCO4256 | - | 17% coverage / 7 total unique peptide | 22% coverage / 9 total unique peptide |
| SCO4257 | - | - | - |
| SCO4258 | - | - | - |
| SCO4259 | Cis15 | - | - |
| SCO4260 | Cis16 | 20% coverage / 4 total unique peptide | 17% coverage / 3 total unique peptide |
