# Supplementary material for: Function and firing of the Streptomyces coelicolor contractile injection system requires the membrane protein CisA
Source: eLife. 2025 Jul 8;14:RP104064. doi: 10.7554/eLife.104064 (PMC12237407; doi:10.7554/eLife.104064)
Supplement: Figure 3—figure supplement 1—source data 1. [file elife-104064-fig3-figsupp1-data1.docx]

**Figure 3-figure supplement 1-source data 1.** Cryo-EM data statistical analysis

|  | Cap | Baseplate_C6 (overall) | Baseplate_C3 |
| --- | --- | --- | --- |
| **Data collection and processing** |  | | |
| Nominal magnification | 81,000 | | |
| Voltage (kV) | 300 | | |
| Electron exposure (e^-^/Å) | ~60 (K3 camera) | | |
| Defocus range (µm) | 1.0 - 3.0 | | |
| Pixel size (Å/pixel) | 1.065 | | |
| Symmetry imposed | C6 | C6 | C3 |
| Initial particles (No.) | 36,569 | 43,087 | 36,569 |
| Final particles (No.) | 19,218 | 22,920 | 18,124 |
| Map resolution (Å) | 3.4 | 3.5 | 3.8 |
| FSC threshold | 0.143 | | |
|  | | | |
| **Refinement** |  | | |
| Model composition |  |  |  |
| Atoms | 33006 (Hydrogens: 243) | 141138 (Hydrogens: 0) | 14343 (Hydrogens: 0) |
| Protein residues | 4266 | 18408 | 1923 |
| Chains | 18 | 60 | 3 |
| R.M.S deviations |  |  |  |
| Bond length (Å) | 0.003 | 0.007 | 0.005 |
| Bond angles (˚) | 0.558 | 0.839 | 0.768 |
| Validation |  |  |  |
| MolProbity score | 1.51 | 2.22 | 1.91 |
| Clashscore | 5.77 | 14.01 | 7.86 |
| Rotamer outlier (%) | 0.03 | 0.04 | 0 |
| Ramachandran plot |  |  |  |
| Favored (%) | 96.81 | 89.51 | 92.18 |
| Allowed (%) | 3.19 | 10.46 | 7.82 |
| Outlier (%) | 0 | 0.44 | 0 |
| Masked CC | 0.77 | 0.79 | 0.77 |
