# Supplementary figures and images for: Function and firing of the Streptomyces coelicolor contractile injection system requires the membrane protein CisA
Source: eLife. 2025 Jul 8;14:RP104064. doi: 10.7554/eLife.104064 (PMC12237407; doi:10.7554/eLife.104064)

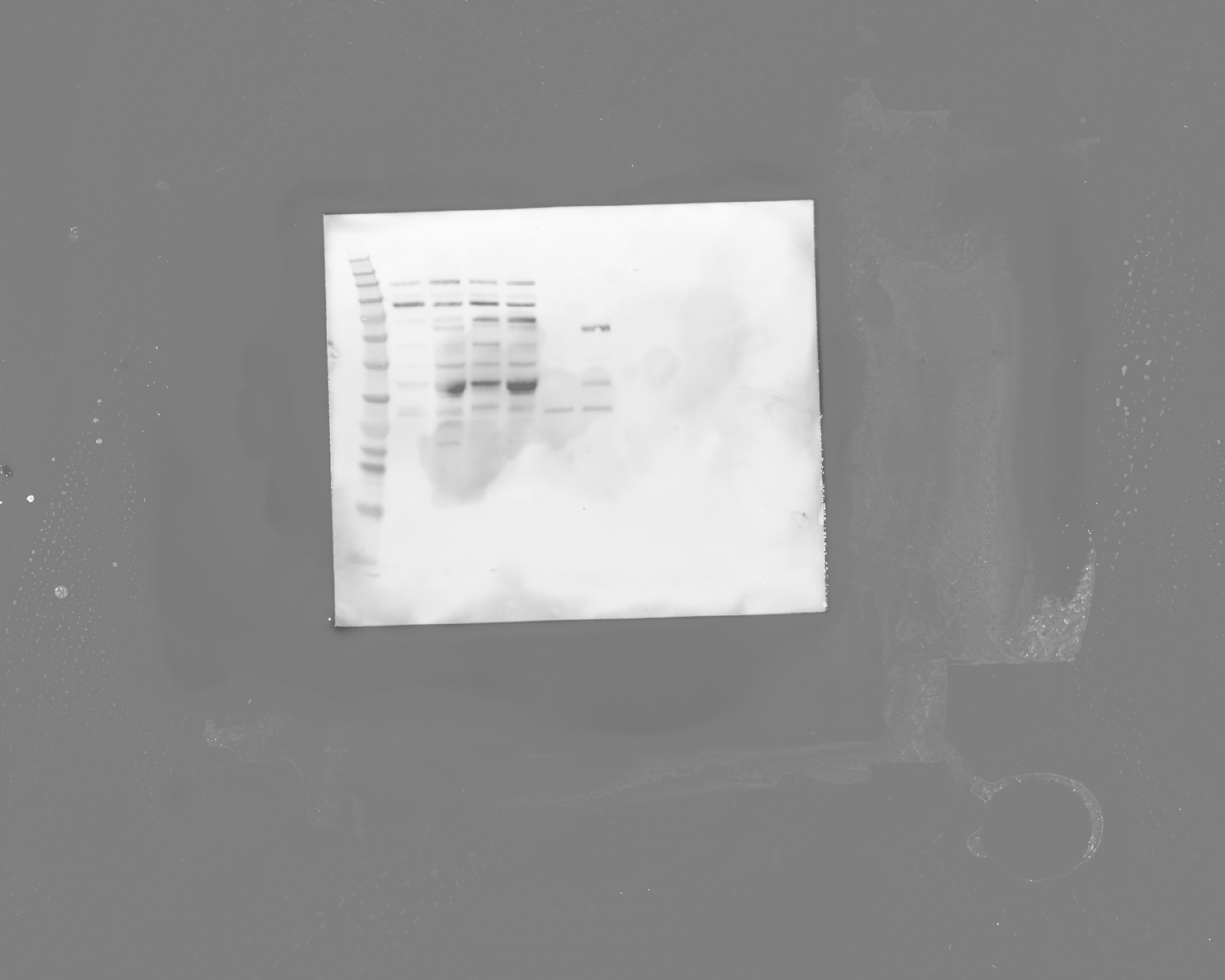

Supplement: Figure 4—source data 3. [file elife-104064-fig4-data3.zip › Figure 4-source data 3/raw_anti-FLAG(Composite).tif]

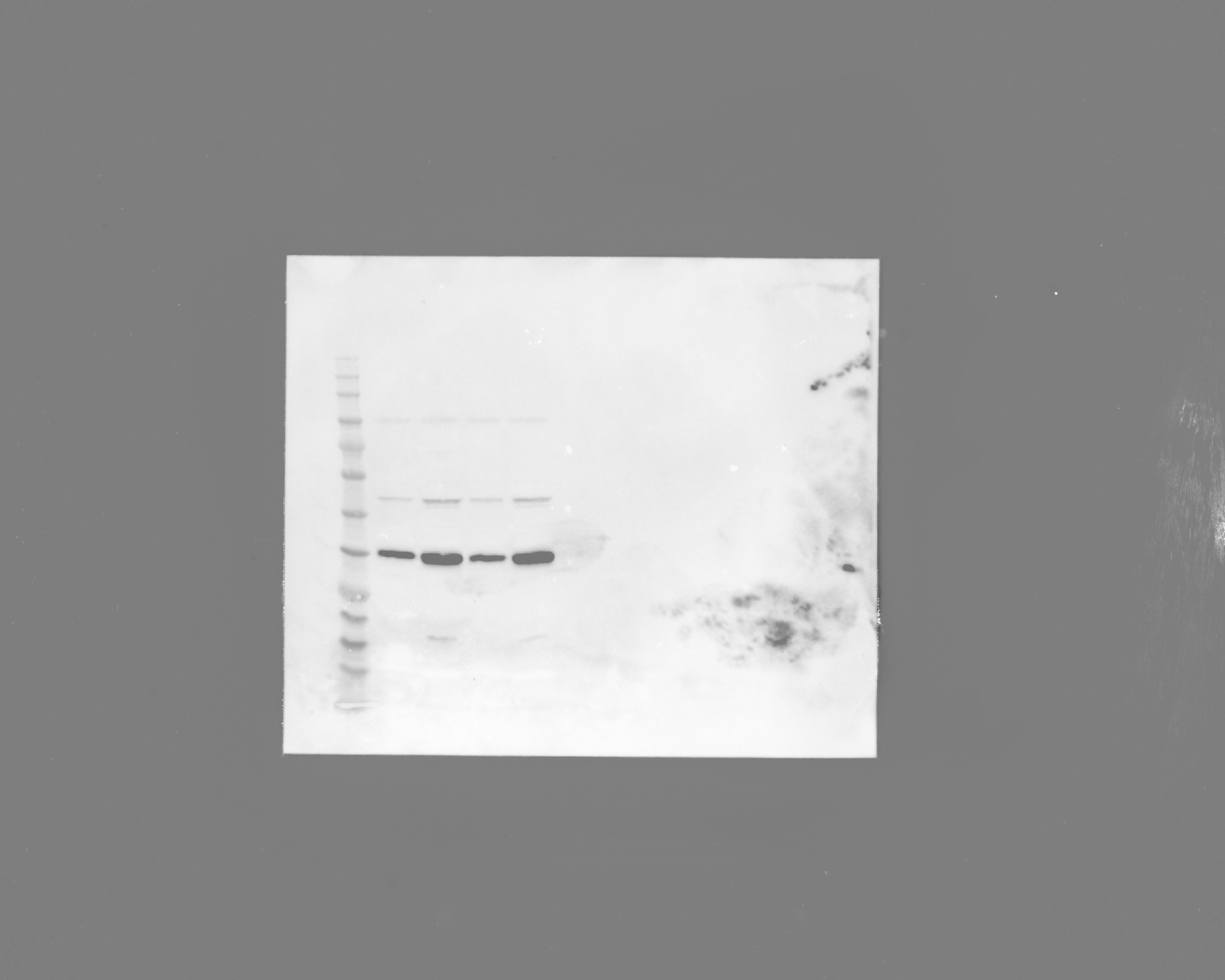

Supplement: Figure 4—source data 3. [file elife-104064-fig4-data3.zip › Figure 4-source data 3/raw_anti-WhiA(Composite).tif]

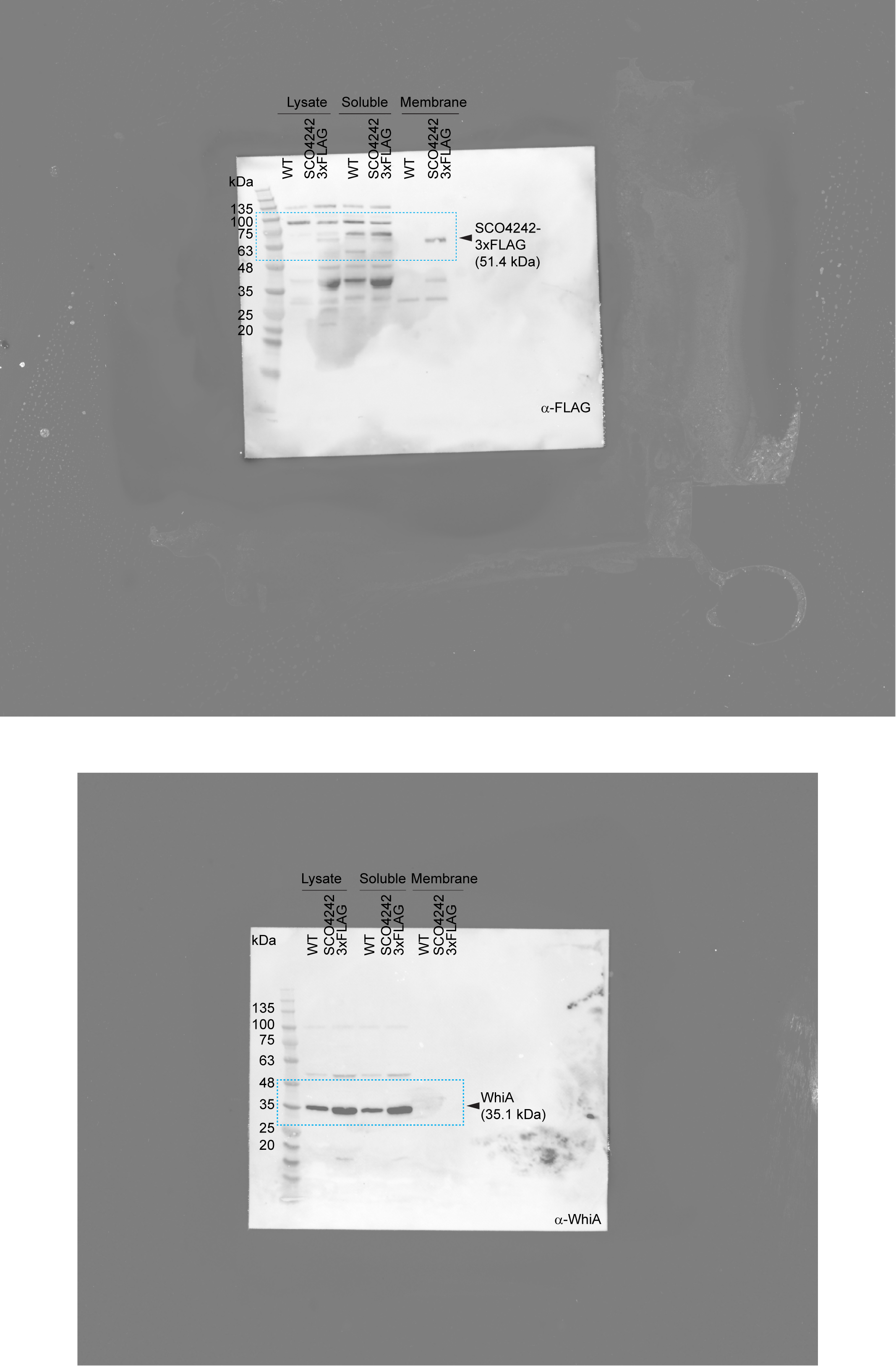

Supplement: Figure 4—source data 4. [file elife-104064-fig4-data4.zip › annotated_raw_immunoblots.tif]

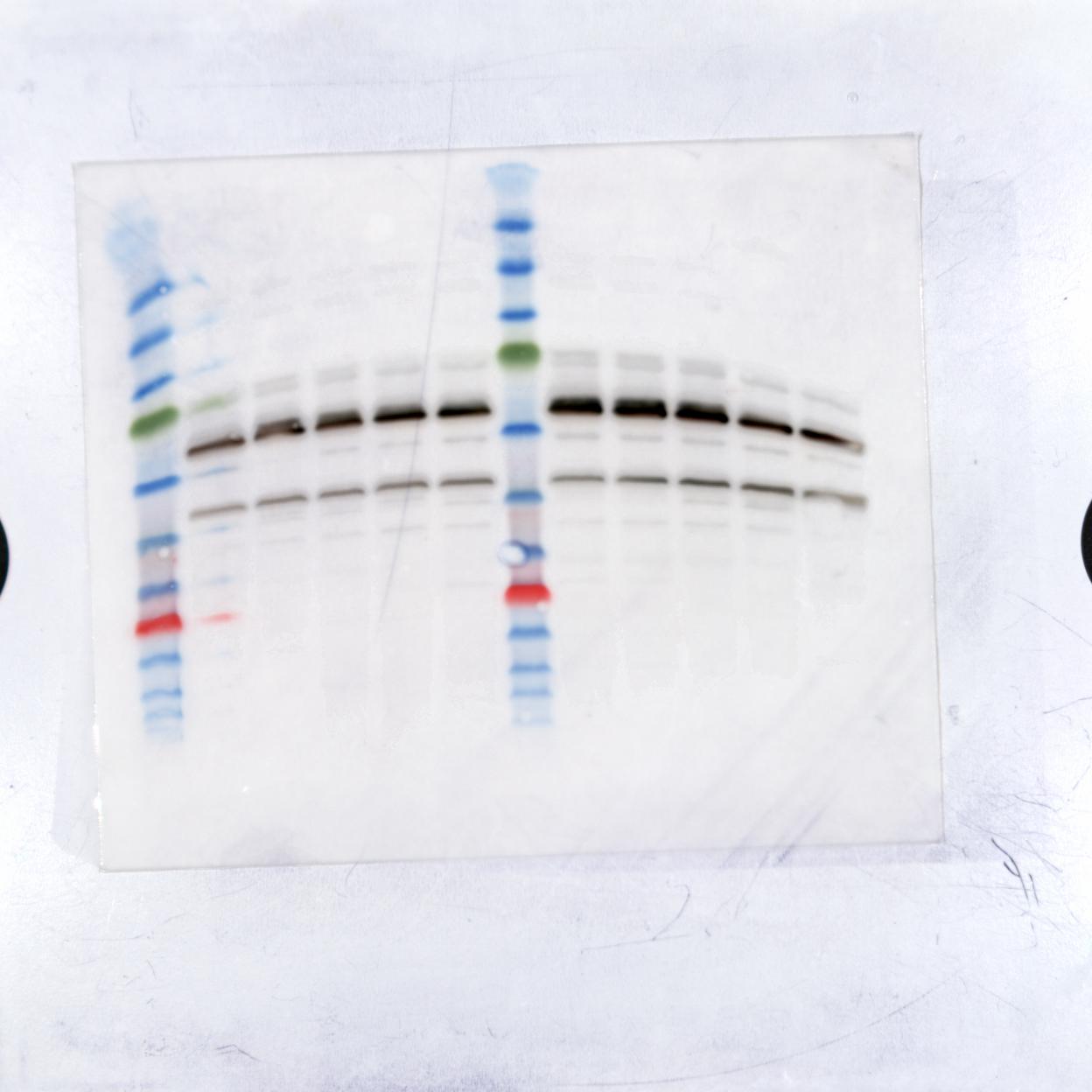

Supplement: Figure 5—figure supplement 3—source data 1. [file elife-104064-fig5-figsupp3-data1.zip › Figure 5-source data 4/anti-GFP(composite).jpg]

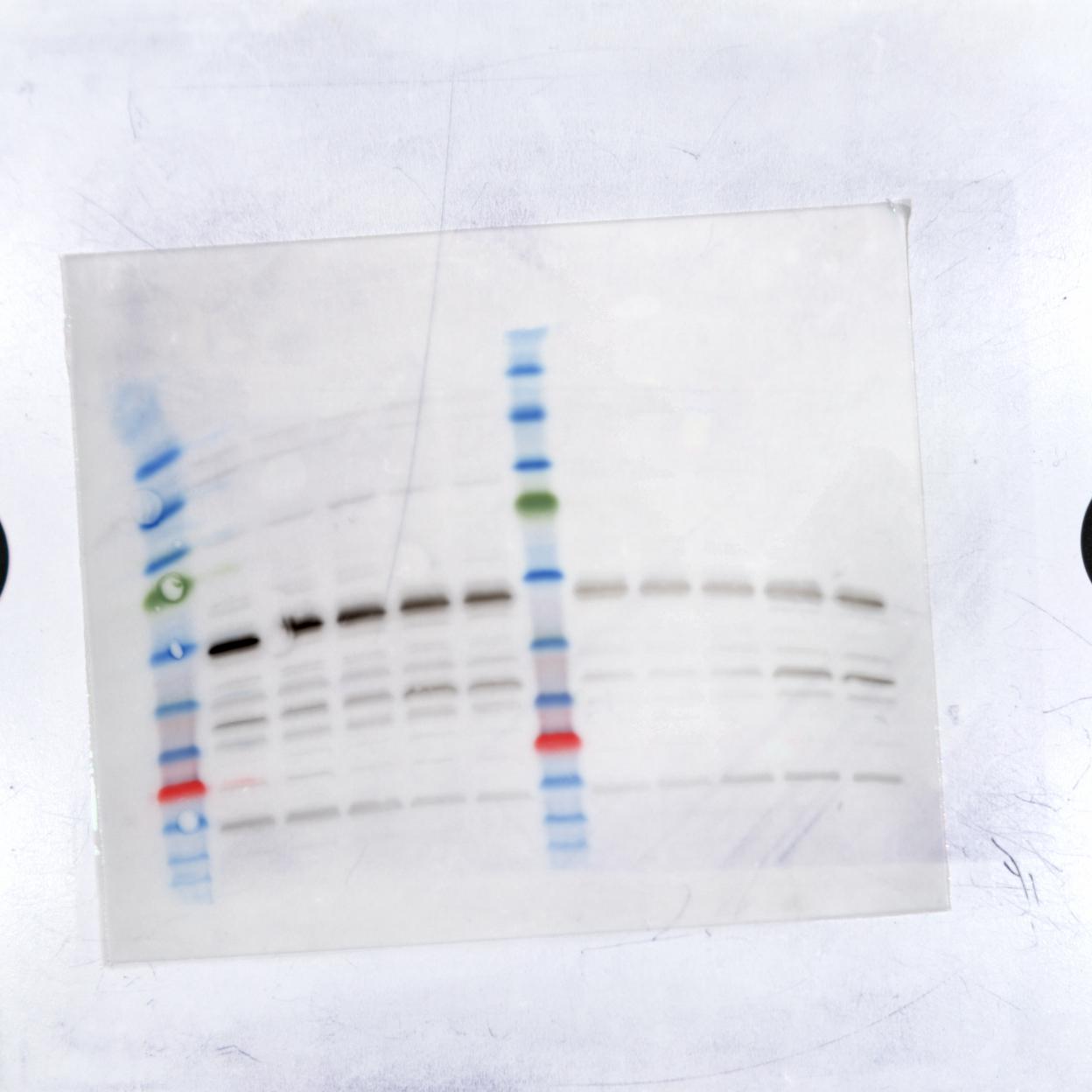

Supplement: Figure 5—figure supplement 3—source data 1. [file elife-104064-fig5-figsupp3-data1.zip › Figure 5-source data 4/anti-WhiA(composite).jpg]

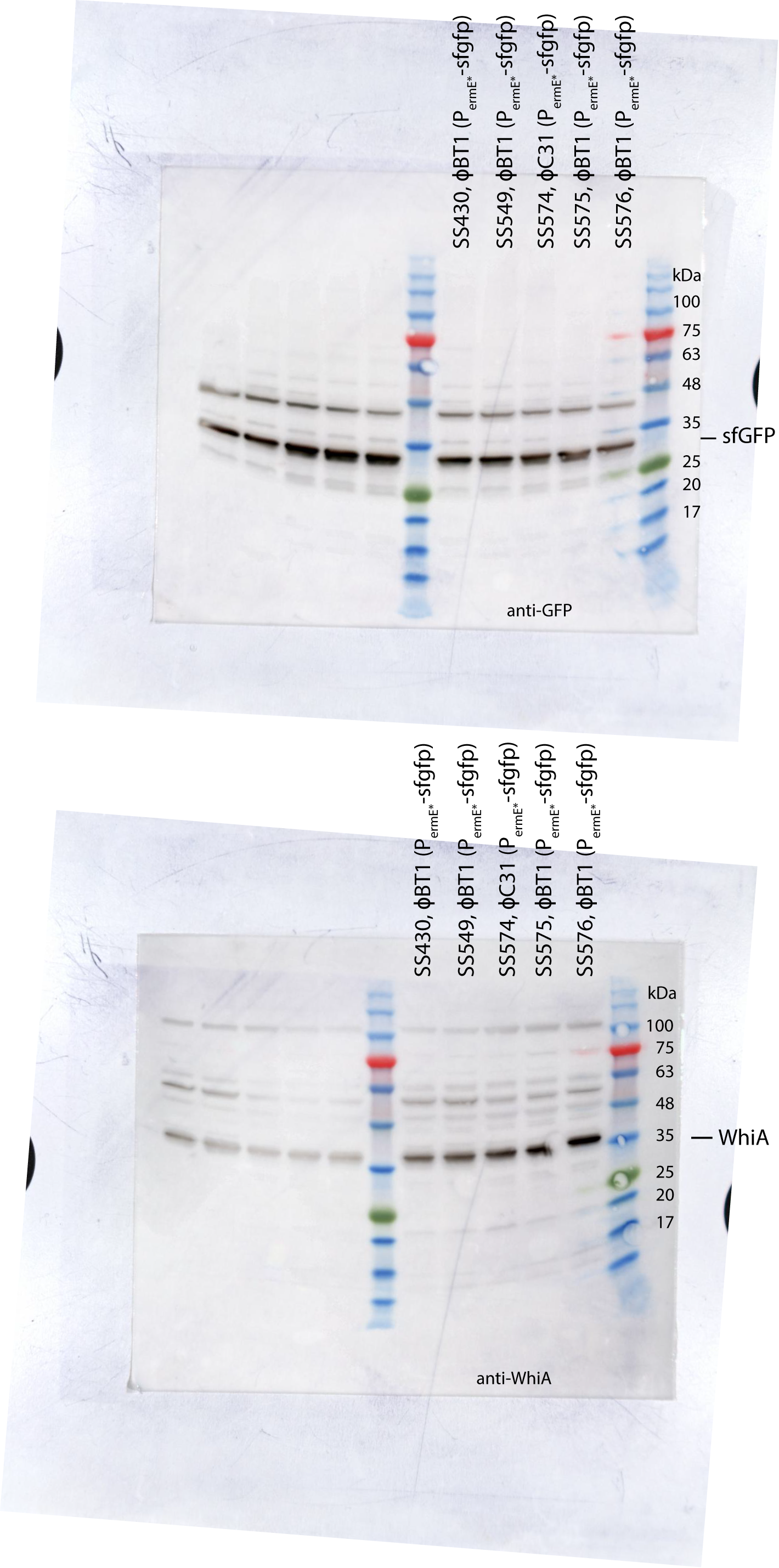

Supplement: Figure 5—figure supplement 3—source data 2. [file elife-104064-fig5-figsupp3-data2.zip › Figure 5-source data 5/Anti-GFP_anti-WhiA_raw+annotated.tif]
