# Supplementary material for: Function and firing of the Streptomyces coelicolor contractile injection system requires the membrane protein CisA
Source: eLife. 2025 Jul 8;14:RP104064. doi: 10.7554/eLife.104064 (PMC12237407; doi:10.7554/eLife.104064)
Supplement: Supplementary file 1. [file elife-104064-supp1.docx]

**Supplementary file 1.** Complete list of strains and plasmids used in this study.

| **Strain** | **Description** | **Construction** | **Source** |
| --- | --- | --- | --- |
| ***Escherichia coli strains*** |  |  |  |
| TOP10 | *F^–^ mcrA Δ(mrr-hsdRMS-mcrBC) Φ80lacZΔM15 ΔlacX74 recA1 araD139 Δ(ara leu) 7697 galU galK rpsL (Str^R^ ) endA1 nupG* | Cloning | Lab strain |
| ET12567/pUZ8002 | *F^–^ dam13::Tn9 dcm6 hsdM hsdR recF143:: Tn10 galK2 galT22 ara-14 lacY1 xyl-5 leuB6 thi-1 tonA31 rpsL hisG4 tsx-78 mtl-1 glnV44* | ET12567 with helper plasmid pUZ8002 | Paget et al., 1999 |
| BW25113/pIJ790 | *Δ(araD-araB)567 ΔlacZ4787(::rrnB-4) lacIp-4000(lacIQ), λ−rpoS369(Am) rph-1 Δ(rhaD-rhaB)568 hsdR514* | BW25113 containing λ RED recombination plasmid pIJ790 | Datsenko and Wanner, 2000 |
| NEB5-alpha | *fhuA2*Δ*(argF-lacZ)U169 phoA glnV44* Φ*80* Δ*(lacZ)M15 gyrA96 recA1 relA1 endA1 thi-1 hsdR17* | Cloning | NEB |
| TG1 | *supE thi-1 Δ(lac-proAB) Δ(mcrB-hsdSM)5 (rK-mK) / F´ traD36 proAB lacIqZΔM15* | Topology Experiment | Lab Strain |
| ***Streptomyces coelicolor strains*** |  |  |  |
| *S. coelicolor* M145 | Wild type (SCP1-, SCP2-) |  | Kieser et al., 2000 |
| SS387 | Δ*SCO4253-51::apr, apr^R^* |  | Casu et al., 2023 |
| SS393 | Δ*SCO4253-51::apr*  *attB φBT1 (SCO4253-N5-*  *SCO4252-51), hyg^R^* |  | Casu et al., 2023 |
| SS430 | WT *ϕBT1 (PermE*-sfgfp)* |  | Casu et al., 2023 |
| SS539 | Δ*SCO4242::apr,*  *apr^R^* | chromosomal *SCO4242 (cisA)* locus deleted using pSS684 | This study |
| SS549 | Δ*SCO4242::apr ϕBT1 (PermE*-sfgfp), hyg^R^, apr^R^* | pSS150 integrated at ΦBT1 attachment site of SS539 | This study |
| SS557 | Δ*SCO4242 φBT1(PermE*-SCO4242-3xFLAG)*  *hyg^R^, apr^R^* | pSS735 integrated at φBT1 attachment site of SS539 | This study |
| SS574 | WT *φC31 (PermE*-sfgfp), apr^R^, thio^R^* | pSS758 integrated at φC31 attachment site of WT | This study |
| SS575 | Δ*SCO4242::apr φC31 (PermE*-sfGFP)*  *apr^R^, thio^R^* | pSS758 integrated at φC31 attachment site of SS539 | This study |
| SS576 | Δ*SCO4242:apr φBT1(PermE*-SCO4242-3xFLAG) φC31 (PermE*-sfGFP)*  *apr^R^, thio^R^, hyg^R^* | pSS758 integrated at φC31 attachment site of SS557 | This study |
| JS65 | Δ*SCO4242:apr φBT1(PermE*-)*  *hyg^R^, apr^R^* | pIJ10257 integrated at φBT1 attachment site of SS387 | This Study |
| JS69 | Δ*SCO4242:apr φBT1(PermE*-SCO4242-mcherry)*  *hyg^R^, apr^R^* | pSS705 integrated at φBT1 attachment site of SS387 | This study |
| **Cosmid and Plasmids** |  |  |  |
| StD8A | Cosmid vector containing  coding sequence for *SCO4242*  km^R^, carb^R^ |  | http://strepdb.  streptomyces.org.uk |
| pIJ773 | pBluescript KS (+) containing the apramycin resistance gene *apr* and *oriT* of plasmid RP4, flanked by FRT sites (Apr^R^). Used as template for the amplification of the *apr-oriT* cassette for ‘REDIRECT’ PCR targeting, apr^R^ |  | Gust et al., 2003 |
| pIJ10257 | Cloning vector for the  conjugal transfer of DNA  (under control of the *ermE**  constitutive promoter).  Integrates at the *ΦBT1* attachment site, hyg^R^ |  | Hong et al., 2005 |
| pKF351 | Derivative of pKF280, integrates at the *ΦC31* attachment site, apr^R^ |  | Schlimpert et al., 2017 |
| pSS150 | pIJ10257 carrying *PermE*-sfgfp*, hyg^R^ |  | Casu et al., 2023 |
| pSS684 | Mutated cosmid StD8A for REDIRECT containing *ΔSCO4242::apr, km^R^, carb^R^, apr^R^* | The *SCO4242* coding sequence on the cosmid vector StD8A was replaced by an *oriT*-containing apramycin resistance cassette, which amplified from pIJ773 using primer 1676/1677 | This study |
| pSS88 | pIJ10257 carrying *mcherry*, *hyg^R^* | *Streptomyces* codon-optimised *mcherry* was inserted between the NdeI-XhoI site of pIJ10257. | This study |
| pSS705 | *PermE*-SCO4242-mcherry, hyg^R^* | *SCO4242* coding sequence amplified with primer 1731/1732 and assembled with pSS88 cut with NdeI-XhoI. | This study |
| pSS734 | *PermE*-SCO4242- TEV-sfgfp, hyg^R^* | A “GGS-TEV” amino acid sequence was added upstream of *sfgfp* from pSS150 by PCR using primer 1800/1801. The PCR product was cut with XhoI-AvrI and inserted into pSS705 cut with the same enzymes. | This study |
| pSS735 | *PermE*-SCO4242-3xFLAG, hyg^R^* | *PermE*-SCO4242* fragment was isolated from pSS705 by restriction digestion with KpnI-XhoI and ligated with pIJ10257 cut with KpnI-XhoI. | This study |
| pSS758 | pKF351 carrying *PermE*-sfgfp, apr^R^, thio^R^* | *sfgfp* amplified from pSS150 with primer 1851/1852 and assembled into pKF351 cut with BamHI-KpnI. | This study |
| **Plasmid used to test membrane topology** |  |  |  |
| pKTop | Plasmid encoding a dual *pho-lac* reporter, km^R^ |  | Karimova et al., 2009 |
| pFRL1 | Plasmid encoding the known *Streptomyces* cytoplasmic protein, SepH, in the dual *pho-lac* reporter, *km^R^* |  | Felix Ramos-Leon (unpublished) |
| pFRL5 | Plasmid encoding the *Streptomyces* membrane protein, RsbN, in the dual *pho-lac* reporter, *km^R^* |  | Felix Ramos-Leon (unpublished) |
| pJUK131 | pKTop carrying *SCO4242, km^R^* | Codon optimized *SCO4242* amplified with primers JS111/JS112 from pJUK123 digested and inserted into *HinDIII*/*Kpn*I digested pKTop | This study |
| pJUK132 | pKTop carrying *SCO4242* (1-310aa), *km^R^* | Codon optimized *SCO4242* containing only codons for aa 1-310 amplified with primers JS111/JS113 from pJUK123 digested and inserted into *HinDIII*/*Kpn*I digested pKTop | This study |
| pJUK145 | pKTop carrying *SCO4242* (1-285aa), *km^R^* | Codon optimized *SCO4242* containing only codons for aa 1-285 amplified with primers JS111/JS114 from pJUK123 digested and inserted into HinDIII/KpnI digested pKTop | This study |
| pJUK146 | pKTop carrying *SCO4242* (Δ295-305aa), *km^R^* | Codon optimized *SCO4242* omitting codons for aa 295-310 amplified with primers JS111/JS112 from pJUK133 digested and inserted into HinDIII/KpnI digested pKTop | This study |
