# Supplementary material for: Function and firing of the Streptomyces coelicolor contractile injection system requires the membrane protein CisA
Source: eLife. 2025 Jul 8;14:RP104064. doi: 10.7554/eLife.104064 (PMC12237407; doi:10.7554/eLife.104064)
Supplement: Supplementary file 2. [file elife-104064-supp2.docx]

**Supplementary file 2**. Oligonucleotides used in this study.

| **Name** | **Sequence (5’🡪3’)** |
| --- | --- |
| 1676 | GGAGAGATGACCACGCAGAACTGCGCCGAGTGCGGAACCATTCCGGGGATCCGTCGACC |
| 1677 | CGGCCCGGGTCAGGAGGAGCGGTTGGCGCTGGACGGGCCTGTAGGCTGGAGCTGCTTC |
| 1691 | atatTCTAGAgATGACCACGCAGAACTGCGCC |
| 1692 | atatGGTACCTCAGGAGGAGCGGTTGGCGC |
| 1693 | atatAAGCTTgATGCCCCTGCCCTCTCCCAAC |
| 1694 | atatGGTACCcgCGCGCTGTCCCCGATCACG |
| 1731 | tggtaggatcgtctagaacaggaggccccatatgACCACGCAGAACTGCGCC |
| 1732 | atgttgtcctcctcgcccttggagaccatctcgagGGAGGAGCGGTTGGCGCT |
| 1767 | agctgtttcctgtgtgaaattgttatcc |
| 1800 | ATATActcgagggtggctccgaaaacctgtacttccaatccatgtccaagggcgaggagc |
| 1801 | aattaaCCTAGGtcacttgtacagctcgtccatgcc |
| 1851 | aagtcgtgctgcttcatgtggtcggggtacccgacccgagcacgcg |
| 1852 | attactggaccggatgaattcacttggatccgttaattaatcactcgagctccgggcccg |
| 1930 | ccaagcttgcatgcctgcagg |
| 1931 | tcacacaggaaacagctatgACCACGCAGAACTGCGCC |
| 1932 | ggcatgcaagcttggGATGCGGTCGAAGACGCGGC |
| 1933 | ccaagcttgcatgcctgcagg |
| 1934 | ggcatgcaagcttggGATGCGGTCGAAGACGCGGC |
| JS105 | CCTCCGCGAATCGGTCGTCTCTCGAGGGTGGCTCCGAAAACCT |
| JS106 | GCGCAATTTTGGGTCGTCATATGTATATCTCCTTCTTAAAGTTAAACAAAATTATTTCTAGAGGGGAA |
| JS107 | TTTAAGAAGGAGATATACATATGACGACCCAAAATTGCGCCGAATGT |
| JS108 | TTTTCGGAGCCACCCTCGAGAGACGACCGATT |
| JS111 | CATTAGAAGCTTTATGACGACCCAAAATTGCGCCG |
| JS112 | CATTAGGGTACCGACGACCGATTCGCGGAGG |
| JS113 | CATTAGGGTACCTGAACCCCGTCGGGAATATA |
| JS114 | CATTAGGGTACCCCGATTCGATCGAAAACACGACGC |
